# Oxytocin receptors in the dorsolateral bed nucleus of the stria terminalis (BNST) bias fear learning toward temporally predictable cued fear

**DOI:** 10.1038/s41398-019-0474-x

**Published:** 2019-04-18

**Authors:** Daisy Martinon, Paulina Lis, Alexandra N. Roman, Patricio Tornesi, Sarah V. Applebey, Garrett Buechner, Valentina Olivera, Joanna Dabrowska

**Affiliations:** 1 Department of Cellular and Molecular Pharmacology, Chicago Medical School, Rosalind Franklin University of Medicine and Science, North Chicago, IL 60064 USA; 2 Center for the Neurobiology of Stress Resilience and Psychiatric Disorders, Rosalind Franklin University of Medicine and Science, North Chicago, IL 60064 USA; 3 School of Graduate and Postdoctoral Studies, Rosalind Franklin University of Medicine and Science, North Chicago, IL 60064 USA

**Keywords:** Long-term memory, Depression

## Abstract

The inability to discriminate between threat and safety is a hallmark of stress-induced psychiatric disorders, including post-traumatic stress disorder. Dorsolateral bed nucleus of the stria terminalis (BNST_dl_) is critically involved in the modulation of fear and anxiety, and has been proposed to regulate discrimination between signaled (cued, predictable) and unsignaled (unpredictable) threats. We recently showed that oxytocin receptors (OTRs) in the BNST_dl_ facilitate acquisition of cued fear measured in a fear-potentiated startle (FPS). In the current study, using in vivo microdialysis in awake male Sprague–Dawley rats, a double immunofluorescence approach with confocal microscopy, as well as retrograde tracing of hypothalamic BNST-projecting OT neurons, we investigated whether fear conditioning activates OT system and modulates OT release. To determine the role of OTR in fear memory formation, we also infused OTR antagonist or OT into the BNST_dl_ before fear conditioning and measured rats’ ability to discriminate between cued (signaled) and non-cued (unsignaled) fear using FPS. In contrast to acute stress (exposure to forced swim stress or foot shocks alone), cued fear conditioning increases OT content in BNST_dl_ microdialysates. In addition, fear conditioning induces moderate activation of OT neurons in the paraventricular nucleus of the hypothalamus and robust activation in the supraoptic and accessory nuclei of the hypothalamus. Application of OT into the BNST_dl_ facilitates fear learning toward signaled, predictable threats, whereas blocking OTR attenuates this effect. We conclude that OTR neurotransmission in the BNST_dl_ plays a pivotal role in strengthening fear learning of temporally predictable, signaled threats.

## Introduction

Oxytocin (OT) is a hypothalamic peptide, hormone, and a neuromodulator, first isolated and then synthesized by Vincent du Vigneaud^1^, who later received Nobel Price for his work. OT receptor (OTR) in a G-protein-coupled receptor, which can propagate signal transduction via either Gα_i_ or Gα_q_ proteins, activate a variety of signaling cascades^2^. In addition to regulating reproductive function and water/electrolyte homeostasis, OT modulates a wide range of fear and anxiety-like behaviors; for review, see refs. ^2,3^. Although substantial evidence suggests that OT has anxiolytic properties^4–6^, the role of OT neurotransmission in the regulation of conditioned fear appears more complex and is brain region specific^7–9^. Some conflicting data on the role of OT in the regulation of fear responses might stem from the fact that the great majority of behavioral studies utilize exogenous OT application to define its biological function, whereas the role of endogenous OT in anxiety and fear formation is largely unknown. In a fear-potentiated startle (FPS), systemic OT reduces background anxiety without affecting cued or contextual fear^10,11^. In the FPS, cued fear is measured as a potentiation of the startle amplitude to startle-eliciting noise during presentations of conditioned stimuli (CS^+^), which have been previously paired with foot shocks. Background anxiety (non-cued fear) reflects potentiation of the startle measured between the CS^+^ presentations. Importantly, non-cued fear recall depends on the initial CS^+^ presentation, as it is not observed until after the CS^+^ presentations^12^, and is mainly independent of contextual fear^10,11^. Therefore, cued and non-cued fear responses can be used to determine rats’ ability to discriminate between signaled (cued) and unsignaled (diffuse) stimuli, as described before^13^.

The dorsolateral bed nucleus of the stria terminalis (BNST_dl_) is a key brain area for translating stress into sustained anxiety^14–16^. Imaging studies in humans have shown potentiation of the BNST activity in conditions of uncertainty^17^, during hypervigilant threat monitoring^18^, and in anticipatory anxiety in participants suffering from arachnophobia^19^. The activity of the BNST is further exaggerated in patients suffering from anxiety disorders^17,18^. In animal models, BNST lesions disrupt expression of contextual fear^20^, as well as conditioned fear responses to long-lasting cues^16,21^, but not to short, discrete cues^22–24^. However, growing evidence suggests that the BNST is also involved in the modulation of conditioned fear responses to discrete cues^25,26^. BNST lesion improves ability to discriminate between cues paired with unconditioned stimuli (US) vs. unpaired cues^27^. Recent studies confirm the involvement of the BNST in learning to discriminate between CS representing safety and CS representing threat^28^, phasic vs. sustained fear^29^, and signaled vs. unsignaled threats^13,25^.

The BNST has one of the highest expression levels of OTR^30–33^ and receives OT inputs, at least partly, from the paraventricular nucleus of the hypothalamus (PVN)^7,30^. We recently demonstrated that OTR neurotransmission in the BNST_dl_ facilitates the acquisition of conditioned fear to a discrete cue^12^. Here we show that OT is selectively released in the BNST_dl_ during cued fear conditioning, highlighting the involvement of endogenous OT in cued fear learning. Moreover, we demonstrate that fear conditioning induces robust activation of OT neurons in the accessory (AN) and supraoptic nuclei of the hypothalamus (SON), and that both of the nuclei project to the BNST_dl_. Finally, using in vivo pharmacology and FPS, we calculate discrimination indices of individual rats by comparing a proportion of cued with non-cued fear as before^13^. We show that OTR transmission in the BNST_dl_ facilitates discrimination learning between cued (signaled, predictable) and non-cued (unsignaled, unpredictable) fear, whereas blocking OTR attenuates this discrimination. Our results show that OTR in the BNST_dl_ biases fear learning toward the formation of adaptive fear responses of cued, signaled, predictable threats.

## Methods and materials

### Animals

Male Sprague–Dawley rats (Envigo, Chicago, IL; 240–300 g) were housed in groups of three on a 12 h light/dark cycle with free access to water and food. Protocols for animal experiments in this study were performed in accordance with the guidelines of the National Institute of Health and approved by the Animal Care and Use Committee at Rosalind Franklin University of Medicine and Science.

### The effect of fear conditioning, acute stress, or social interactions on OT content in BNST_dl_ microdialysates

#### Fear conditioning

A total of 31 rats were used in the experiment (Supplementary Table 1), 6 rats were eliminated due to missing microdialysate samples, and 2 rats were eliminated due to misplacement of the probe. Microdialysis in freely moving rats was performed as before^34^ (Supplementary Methods 1.1-1.2).

**Fig. 1:**
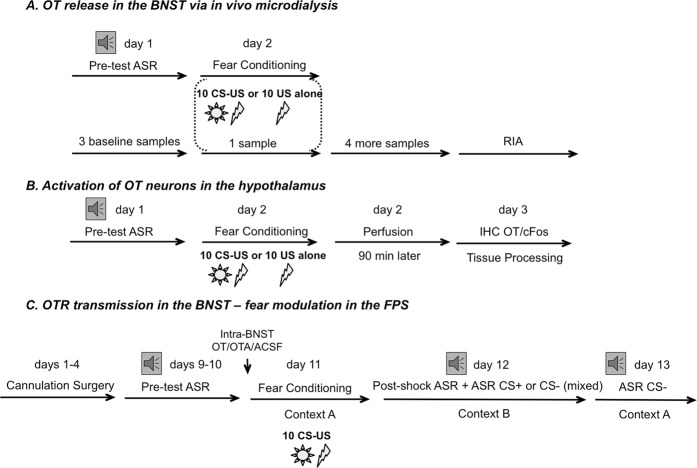
Schematic representation of the experimental design (**a**–**c**). **a** Rats were habituated to the chambers and tested for an acoustic startle response (pre-test ASR). On the following day, BNST_dl_ microdialysates were collected prior, during, and following fear conditioning. Rats were subjected to either cue lights paired with foot shocks (CS-US), or unsignaled foot shocks (US alone). Control rats were placed inside the microdialysis cages without a light or shock exposure. **b** Following exposure to foot shocks signaled by a cue, unsignaled foot shocks, or control conditions (exposure to cue alone or no cue, no foot shock exposure) rats were perfused and hypothalamic sections were processed for double immunofluorescence labeling with antibodies against OT and marker of immediate early gene expression, cFos. **c** Before cued fear conditioning (CS-US), cannulated rats were infused bilaterally into the BNST_dl_ with OT, oxytocin receptor antagonist (OTA), or artificial cerebrospinal fluid (ACSF) in context A. Twenty-four hours later, rats were tested for the recall of cued and non-cued fear in context B. The recall test consisted of 10 habituation ASR trials (excluded from analysis), followed by ASR measured during the presence (CS^+^) or absence of cue light, mixed in a pseudorandom order (40 trials). Twenty-four hours later, rats were tested for the contextual fear recall (ASR measured without CS^+^ presentations) in context A

All experiments were conducted in SR-LAB startle chambers (San Diego Instruments, San Diego, CA)^12^ (Supplementary Methods 1.3.1). On any experimental day, baseline microdialysis samples were collected from three rats. After 5 min of acclimation in SR-LAB chambers, one experimental rat received ten presentations of a 3.7 s cue light (CS), each co-terminating with a 0.5 s foot shock (US; 0.5 mA, inter-trial interval 60–180 s). Another rat placed in a neighboring chamber received the same ten foot shocks without the CS. Samples from the third control rat, placed in a Plexiglas microdialysis cage, were continuously collected in the same room (Fig. 1a). After three baseline microdialysis samples’ collection, fear-conditioning session began in parallel with the fourth sample collection. Session started with 5 min acclimation, continued for 20 min, and the rats remained inside the chambers for additional 5 min after the session ended, after which the microdialysate sample was collected (100 μl). The rats were then returned to their microdialysis cages and four more samples were collected in 30 min intervals (100 μl each).

#### Forced swimming

There were 41 rats used in the forced swimming (FS) and social interaction (SI) experiments, and 9 rats were excluded from the analysis (misplacement or inability to confirm probe placement). After baseline samples collection, rats were placed in Plexiglas tanks filled with water for 10 min^35^. They were then returned to microdialysis cages and five more microdialysis samples were collected (Supplementary Methods 1.3.2).

#### Social interactions

After three baseline sample collections, a novel rat was placed in the microdialysis cage with the experimental rat for 10 min (Supplementary Methods 1.3.3).

#### Probe placement and radioimmunoassay for OT

**Fig. 2:**
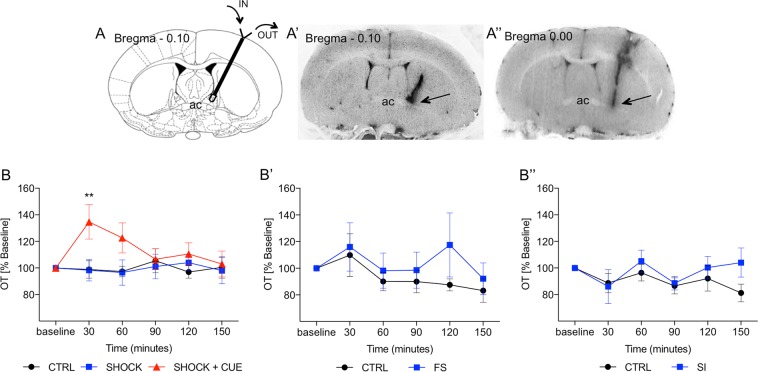
Fear conditioning, but not acute stress, evokes OT release in the BNST (**a**, **b**”). Cued fear conditioning evokes OT release in the BNST_dl_. **A-A**” Representative brain sections with a unilateral track of a microdialysis probe. Upon completion of the experiments, the probes were perfused with Chicago Sky Blue 6B dye. All extracted brains were sliced and all BNST sections were photographed to confirm proper placement of the probe. Examples of confirmed probe locations in the BNST_dl_ (Bregma + 0.10 mm to −0.36 mm), which met the following criteria: above the anterior commissure (ac), below the lateral ventricle, and medially to the internal capsule as indicated by the arrows (included in the analysis: **A**’ unilateral probe hit). Examples of misplaced probe locations with probe track too lateral to the BNST_dl_ as indicated by the arrow (**A**”, excluded from the analysis). **B-B**” Exposure to foot shocks signaled by a cue, but not unsignaled foot shocks, increases OT content in BNST_dl_ microdialysates. Two-way repeated-measures ANOVA revealed a significant interaction between TIME and TREATMENT (*P* = 0.0408), and post-hoc analysis with Bonferroni’s showed a significantly greater percentage change of OT content in BNST_dl_ microdialysates in rats exposed to cue-signaled foot shocks (134.66% ± 12.95 of baseline content) at 30 min in comparison with CTRL rats (98.86% ± 6.56, **P < 0.01) as well as in comparison with rats exposed to unsignaled foot shocks (98.29% ± 8.04, **P < 0.01) (**b**). In contrast, forced swim stress (FS; **B**’) or social interactions (SI; **B**”) did not affect OT content in BNST_dl_ microdialysates

Radioimmunoassay was performed as before^34^ (see inclusion criteria in Fig. 2 legend and Supplementary Methods 1.4-1.5).

### The effect of fear conditioning on activation of hypothalamic OT neurons

#### Fear conditioning and timely perfusions

Ninety minutes following exposure to unsignaled foot shocks, foot shocks signaled by a cue, or control conditions (*n* = 5 per condition), rats were deeply anesthetized with Somnasol (Henry Schein Animal Care, Dublin, OH) and transcardially perfused with 10% buffered formalin. In another cohort, rats were exposed to cue light alone or control conditions (*n* = 3 per condition) and were perfused as above. Brains were then sliced (50 μm) on a SM2000R sliding microtome (Leica Biosystems, Nussloch, Germany) and processed for double immunofluorescence for OT and immediate early gene expression, cFos^30^ (Fig. 1b, Supplementary Methods 2.1).

#### Confocal microscopy and cells counting

An FV10i confocal laser-scanning microscope (Olympus, Waltham, MA) was used to acquire high-resolution *Z*-stack images (×60 magnification at 1 μm interval), which were taken bilaterally from the PVN, SON, and AN (both medial and lateral AN when applicable) of every third hypothalamic brain section from the entire brain (150 μm interval between sections, five to six sections from each rat; Supplementary Table 2). Quantitative analysis of double-labeled OT/cFos neurons was performed offline from all *Z*-stacks from the three-hypothalamic nuclei using FLUOVIEW software (Version 3.0, Olympus) by experimenters, who were blind to the treatments during image acquisition as well as during cell counting. For each *Z*-stack image, the cells colocalizing cFos and OT were counted and compared with the total number of neurons expressing OT alone (Supplementary Methods 2.2).

### Retrograde tracing of BNST-projecting hypothalamic OT neurons

Six rats were bilaterally injected into the BNST_dl_ with 200 nl (25 nl/min) of retrograde tracer, cholera toxin B Alexa Fluor 488 conjugate (CTB, Life Technologies, C34775, Eugene, OR). Rats were transcardially perfused 4 weeks later and hypothalamic sections were immunolabeled for OT as above. Imaging was completed using Nikon A1R and Olympus FV10i confocal microscopes.

### The effects of OT or OTA administration into the BNST_dl_ on the FPS

Guide cannulas were implanted bilaterally into the BNST_dl_ of 75 rats as before^26^. Twenty-three artificial cerebrospinal fluid (ACSF)-treated, 14-OT, and 16-OTR antagonist (OTA)-treated rats were included in the main analysis. Ten OT- and 12 OTA-treated rats were excluded from the analysis due to cannula misplacement and inability to confirm the location of the cannula (see inclusion criteria in Supplementary Materials 1.4) and were analyzed as negative controls (Fig. 5A-A”’).

#### Drugs

OT acetate salt (H-2510, Bachem, Inc., CA) and a selective OTA (V-905, NIMH Chemical Synthesis and Drug Supply Program) (d(CH_2_)_5_^1^, D-Tyr^2^, Thr^4^, Orn^8^, des-Gly-NH_2_^9^)-vasotocin trifluoroacetate salt^36^ were stored in −80 °C freezer and diluted in sterile ACSF (pH = 7.4) before each experiment (Supplementary Methods 3.2).

#### Fear conditioning and fear recall testing using FPS

FPS procedures were performed as before^10–12,37^ (Supplementary Methods 3.3). On days 1 (startle habituation) and 2 (pre-test), rats underwent two acoustic startle response (ASR) sessions, they were fear conditioned in context A on day 3, and 24 h later rats were tested for cued and non-cued fear expression (recall test, day 4) in context B. On day 5, the same rats were tested for contextual fear recall in context A (Fig. 1c).

#### Data analysis

FPS analysis, including percentage change of cued and non-cued fear, was performed as before^12^ (Supplementary Methods 3.4). The following formulas were used: Cued fear = [(light-noise trials − noise-alone trials)/noise-alone trials] × 100% in context B. Non-cued fear = [(noise-alone trials − pre-test trials)/pre-test trials] × 100% in context B. Contextual fear = [(noise-alone trials − pre-test trials)/pre-test trials] × 100% in context A. In addition, to determine the ability to discriminate between cued and non-cued fear, we calculated the discrimination index (DI) of individual rats by dividing their percent change score of cued fear by their percent change score of non-cued fear responses according to the formula: DI = [(light-noise trials/noise-alone trials)/(noise-alone trials/pre-test trials)] in context B.

### Statistical analysis

Data are presented as mean ± SEM. In the microdialysis experiments, results (pg/100 μl) were analyzed by a within-group, one-way repeated-measures (RM) analysis of variance (ANOVA) (Supplementary Table 1). For analysis between treatment groups, data are presented as percentage change (±SEM) from the subjects’ own baseline values and analyzed by a two-way RM ANOVA with the factors TIME and TREATMENT. ANOVA was used to compare percentages of OT neurons colocalizing cFos in each of the hypothalamic nuclei between three conditions (TREATMENT; Supplementary Table 2). FPS data were analyzed by a two-way RM ANOVA with the factors TRIAL TYPE (pre-test, noise-alone, light-noise) and TREATMENT. The percent change scores (cued, non-cued, and contextual fear), shock reactivity, and DI were analyzed using ANOVA. To determine the effect of treatment on DI scores as a function of time, results were analyzed with a two-way RM ANOVA with the factors TIME (recall session divided into four blocks, each consisting of five noise-alone and five light-noise trials) and TREATMENT. Where the F-ratio was significant, all pairwise post-hoc comparisons were made using Bonferroni’s test. All statistical analyses were completed using GraphPad Prism version 6 (GraphPad Software, Inc., San Diego, CA). *P* < 0.05 was considered significant.

## Results

### The effects of behavioral manipulations on OT content in BNST_dl_ microdialysates

OT content (pg/100 μl) in BNST_dl_ microdialysates is shown in Supplementary Table 1.

#### Cued fear conditioning increases OT content in BNST_dl_ microdialysates

To determine whether OT levels were stable before introducing fear conditioning, we performed RM ANOVA of OT content in three baseline samples and have found stable OT levels in rats exposed to foot shocks alone (F(1.150,5.751) = 1.977, *P* = 0.2148), foot shocks signaled by a cue (F(1.502,10.51) = 1.740, *P* = 0.2216), and control (CTRL) conditions (F(1.106,6.636) = 1.331, *P* = 0.2948).

To determine whether fear conditioning affected OT content in BNST_dl_ microdialysates within each treatment group, we performed RM ANOVA. This revealed a significant TREATMENT effect on OT content in rats exposed to foot shocks signaled by a cue (F(3.010,21.07) = 3.621, *P* = 0.0297), but not rats exposed to foot shocks alone (F(2.597,12.98) = 0.1707, *P* = 0.8921), or CTRL rats (F(2.502,15.01) = 0.08814, *P* = 0.9468).

A two-way RM ANOVA of percentage change from baseline OT content allowed us to perform comparisons between treatment groups. This analysis showed no significant main effect of TREATMENT (F(2,20) = 1.937, *P* = 0.1702) or TIME (F(5,100) = 1.153, *P* = 0.3378). However, there was a significant interaction between TIME and TREATMENT (F(10,100) = 2.002, *P* = 0.0408). Bonferroni’s multiple comparison test revealed a significantly greater percentage change of OT content in rats exposed to foot shocks signaled by a cue (134.66% ± 12.95) at 30 min in comparison with CTRL rats (98.86% ± 6.56, *P* < 0.01), or in comparison with rats exposed to foot shocks alone (98.29% ± 8.04, *P* < 0.01). No significant effects were observed at 60, 90, 120, and 150 min after the fear conditioning (Fig. 2b).

#### FS does not affect OT content in BNST_dl_ microdialysates

OT content did not differ between baseline microdialysates in rats exposed to FS (F(1.235,8.644) = 1.405, *P* = 0.2780) or CTRL rats (F(1.542,3.88) = 1.014, *P* = 0.3680).

No significant effect of TREATMENT was observed in rats exposed to FS (F(2.275,15.92) = 0.7365, *P* = 0.5109) or CTRL rats (F(2.510,22.59) = 1.220, *P* = 0.3209, RM ANOVA).

Comparing percentage change from baseline OT content revealed no significant main effect of TREATMENT (F(1,16) = 1.010, *P* = 0.3298) or TIME (F(5,80) = 1.414, *P* = 0.2282), and no significant interaction between TIME and TREATMENT (F(5,80) = 0.4828, *P* = 0.7881, two-way RM ANOVA; Fig. 2b**’**).

#### SIs do not affect OT content in BNST_dl_ microdialysates

OT content did not differ between baseline BNST_dl_ microdialysates in SI rats (F(1.115,5.573) = 2.160, *P* = 0.1977) or CTRL rats (F(1.564,9.383) = 1.651, *P* = 0.2403).

No significant effect of treatment on OT content was observed in rats exposed to SI (F(2.371,11.85) = 1.379, *P* = 0.2932) or CTRL rats (F(2.260,15.82) = 1.332, *P* = 0.2949).

Comparing percentage changes from baseline OT content revealed no significant main effect of TREATMENT (F(1,12) = 1.432, *P* = 0.2546), no significant effect of TIME (F(5,60) = 1.394, *P* = 0.2396), and no significant interaction between TIME and TREATMENT (F(5,60) = 0.8411, *P* = 0.5259; Fig. 2b”)

### The effect of fear conditioning on OT neurons’ activation in the hypothalamus

**Fig. 3:**
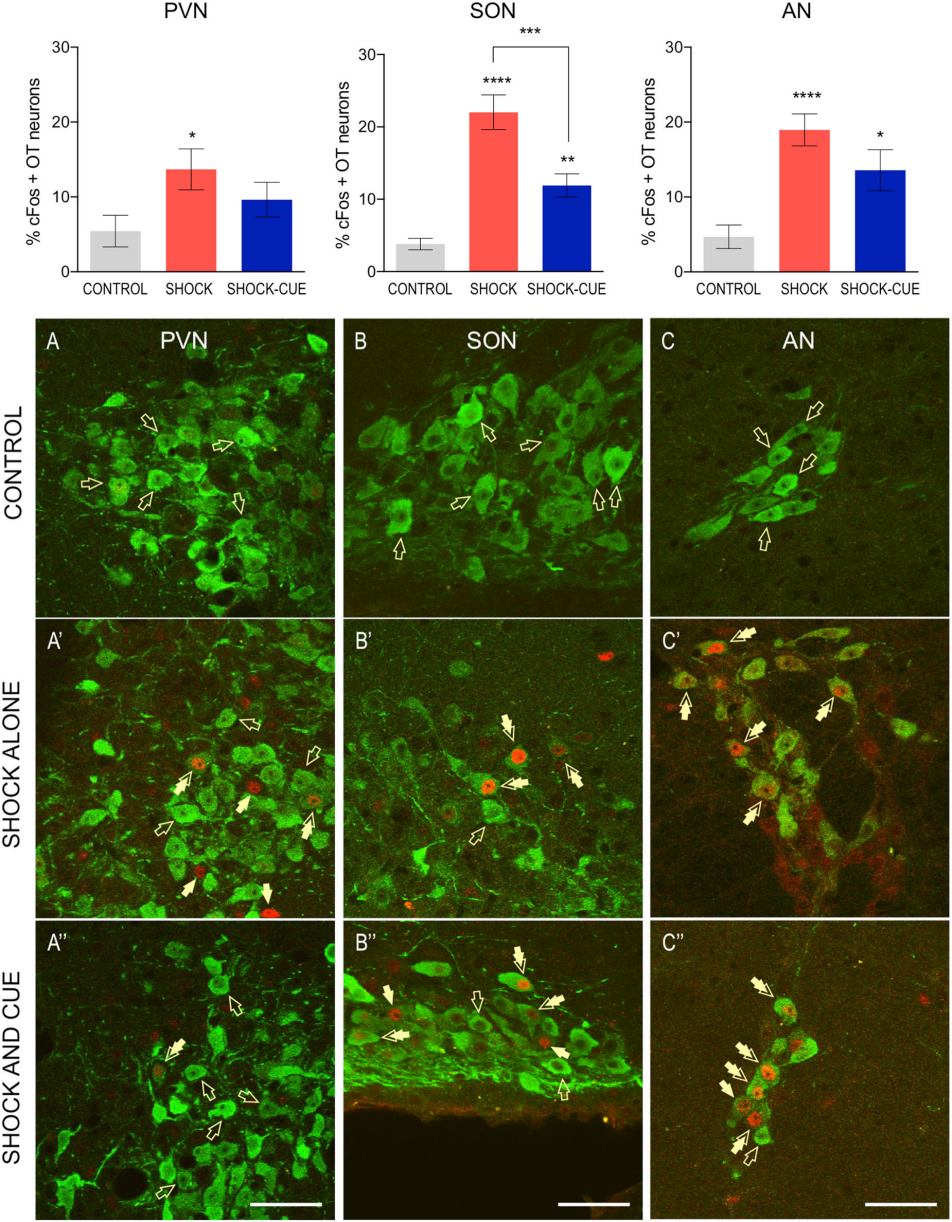
Fear conditioning increases percentage of activated OT neurons in the PVN, SON, and AN. Upper panel: in the PVN, there was a significant effect of fear conditioning on OT neurons activation (*P* = 0.0465), with a significantly greater percentage of activated OT neurons in rats exposed to foot shocks alone in comparison with control rats (*P < 0.05). OT neurons within the SON were significantly activated in response to fear conditioning (*P* < 0.0001, one-way ANOVA), with a greater percentage of OT neurons co-expressing cFos in rats exposed to un-signaled foot shocks compared with control rats (****P < 0.0001), as well as in rats exposed to foot shocks signaled by a cue compared with controls (**P < 0.01). Finally, there was a greater activation of OT neurons in response to foot shocks alone vs. foot shocks signaled by a cue in the SON (***P < 0.001). Fear conditioning activated OT neurons in the AN (****P < 0.0001, one-way ANOVA), with a significantly greater percentage of activated OT neurons in rats exposed to unsignaled foot shocks (****P < 0.0001) and in rats exposed to foot shocks signaled by a cue (*P = 0.0168), compared with control rats. Bottom panel: although control rats show little co-expression of OT (green, open arrows) and cFos (red, closed arrows) in the PVN (**A**), rats exposed to foot shocks alone (**A’**), but not foot shocks signaled by a cue (**A**”), show increase in percentage of OT neurons co-expressing cFos in the PVN. In the SON, number of OT neurons co-expressing cFos was significantly increased in response to unsignaled foot shocks (**B’**) as well as foot shocks signaled by a cue (**B**”), in comparison with control rats (**B**). In the AN, percentage of OT neurons co-expressing cFos was increased in response to foot shocks alone (**C’**) as well as foot shocks signaled by a cue (**C**”), in comparison with control rats (**C**”, magnification ×60, scale bar 50 μm)

Results are shown in Fig. 3. Additional analysis is included in the Supplementary Table 2. The average number of counted OT neurons, including anterior to posterior hypothalamic sections scored bilaterally, was 44.18 ± 3.17 for each hemisphere PVN, 59.62 ± 2.48 for each SON, and 13.62 ± 0.80 for each AN, for all treatment groups combined.

#### Fear conditioning activates OT neurons in the PVN

When comparing percentages of cFos-positive OT neurons in the PVN, ANOVA revealed a significant main effect of TREATMENT in response to fear conditioning (F(2,129) = 3.142, *P* = 0.0465). Post-hoc analysis showed a significantly greater percentage of activated OT neurons in rats exposed to foot shocks alone in comparison with CTRL rats (*P* = 0.0406). However, percentage of activated OT neurons did not differ between rats exposed to foot shocks signaled by a cue compared with CTRL rats (*P* = 0.6495), or compared with rats exposed to foot shocks alone (*P* = 0.7511) (Fig. 3A-A”).

#### Fear conditioning causes robust activation of OT neurons in the SON

Similarly, in the SON, there was a significant effect of TREATMENT on percentage of activated OT neurons in response to fear conditioning (F(2,82) = 31.40, *P* < 0.0001). Post-hoc analysis revealed a significantly greater percentage of activated OT neurons in rats exposed to foot shocks signaled by a cue (*P* = 0.0030) and foot shocks alone (*P* < 0.0001) in comparison with CTRL rats. A significant difference was also observed in rats exposed to signaled vs. unsignaled footshocks (*P* = 0.0003) (Fig. 3B-B”).

#### Fear conditioning causes robust activation of OT neurons in the AN

A significant TREATMENT effect (F(2,150) = 12.62, *P* < 0.0001) followed by a post-hoc analysis revealed a significantly greater percentage of activated OT neurons in rats exposed to foot shocks signaled by a cue (*P* = 0.0168) as well as group exposed to foot shocks alone (*P* < 0.0001), as compared with CTRL. Percentages of activated OT neurons did not differ between the two groups (*P* = 0.2564) (Fig. 3C-C”). Interestingly, the most intense OT-cFOS co-expression was observed in posterior AN sections in both groups, reaching 25% of all OT neurons co-expressing cFos (Bregma −1.80 to −2.28 based on Rat Brain Atlas^38^; Supplementary Table 2.

#### Exposure to cue alone does not activate OT neurons in the hypothalamus

To determine whether exposure to CS alone can cause an activation of OT neurons, percentage of activated OT neurons was compared between rats exposed to cue (light) alone and control rats. In the PVN, the percentage of OT neurons co-expressing cFos did not differ between rats exposed to cue alone and control rats (*t* = 0.5609, df = 45, *P* = 0.5776, unpaired *t*-test). Similarly, in the SON, no treatment effect was detected (*t* = 0.7189, df = 27, *P* = 0.4784). Finally, no cue effect was also observed in the AN (*t* = 1.063, df = 32, *P* = 0.2959).

### OT neurons from the hypothalamus project to the BNST_dl_

**Fig. 4:**
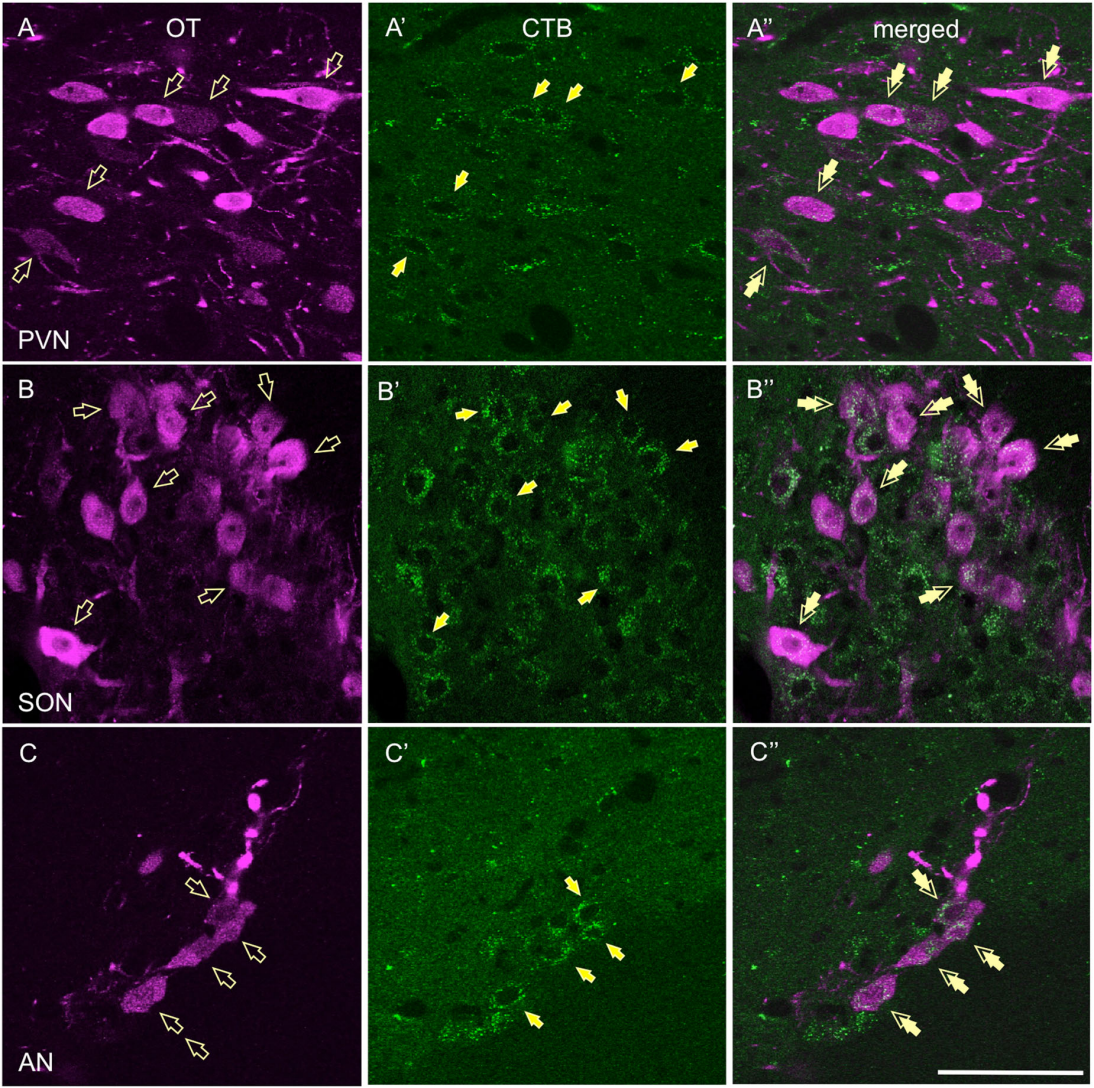
Hypothalamic OT neurons project to the BNST (a-c''). OT neurons from the PVN (**a**), the SON (**b**), and the AN (**c**) project to the BNST_dl_. Four weeks after a retrograde tracer (cholera toxin B, CTB, tagged with Alexa Fluor 488) was infused into the BNST_dl_, CTB-positive neurons were found in the PVN (**A’**), anterior SON (**B’**), and posterior AN (**C’**, green, open arrows). Some of the CTB neurons also co-expressed OT in the PVN (**A**”), the SON (**B**”), and the AN (**C**”, magenta, double arrows, ×60, scale bar 50 μm)

To determine which hypothalamic OT neurons project to the BNST_dl_, we injected a retrograde tracer, CTB, into the BNST_dl_, and several weeks later we stained serial hypothalamic brain sections with OT. Double-labeled neurons for CTB-Alexa 488 and OT were found in the PVN (Fig. 4A-A”), anterior SON (Fig. 4B-B”, Bregma −1.20 to Bregma −1.32 mm), and posterior AN (Fig. 4C-C”), indicative of BNST-projecting OT neurons.

### Effects of OT or OTA administration into the BNST_dl_ on fear acquisition

#### Acquisition of cued fear conditioning

**Fig. 5:**
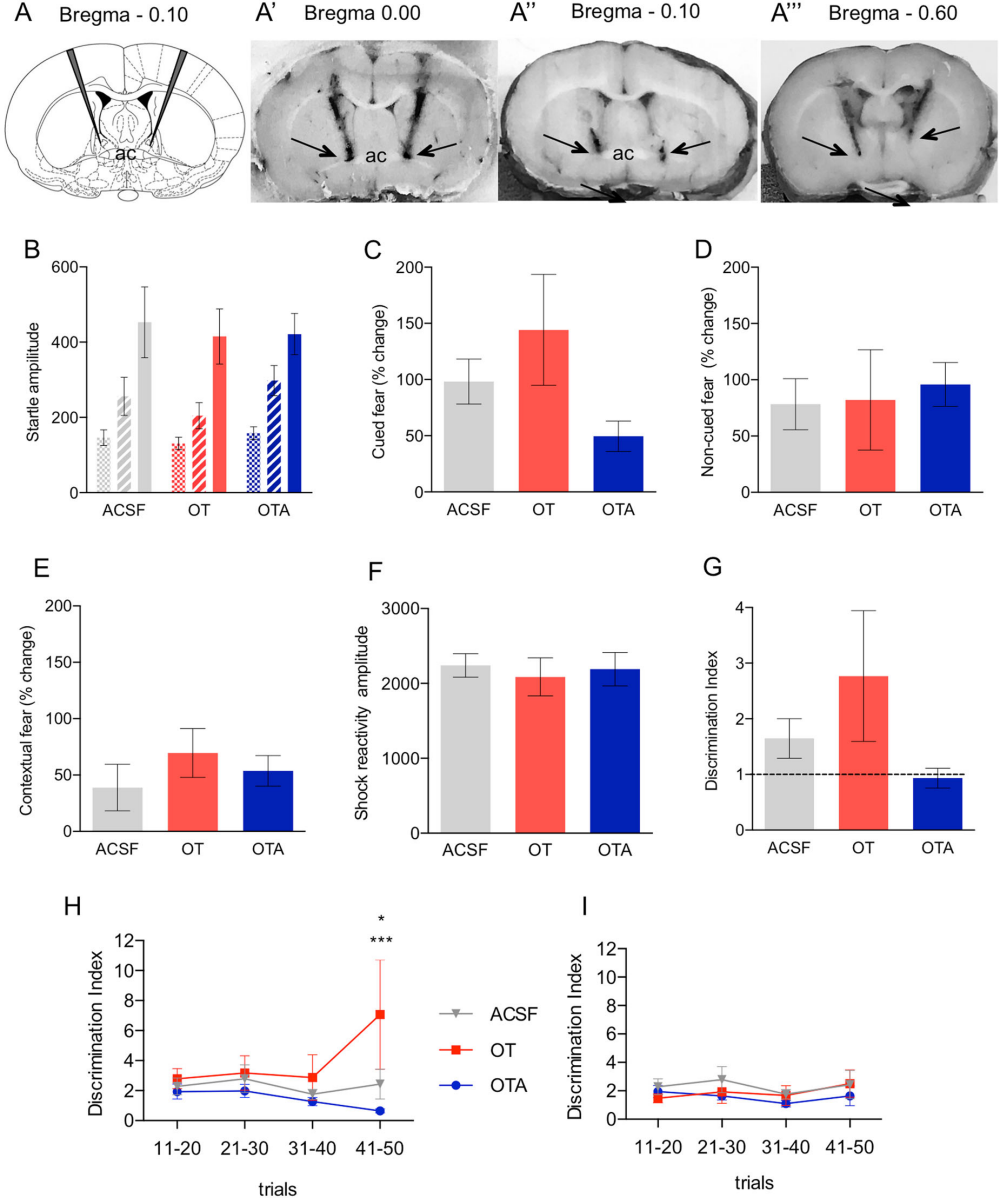
Oxytocin receptors in the BNST facilitate cued fear (**a**–**i**). OTR transmission in the BNST_dl_ facilities formation of cued fear measured in the FPS. **A-A**”’ Representative brain sections with bilateral cannulas targeting the BNST_dl_. Bilateral cannula hit (**A’**), unilateral cannula hit (**A**”), or cannula too posterior to the BNST_dl_ (**A**”’). **b**–**g** The effects of intra-BNST_dl_ administration of ACSF, OT, or OTA on acquisition of fear conditioning measured in the FPS. Group data for pre-test, noise-alone, and light-noise startle amplitude from rats given bilateral intra-BNST_dl_ ACSF (*n* = 23, gray), OT (100 ng, *n* = 14, red), or OTA (200 ng, *n* = 16, blue), 10 min before the fear-conditioning session. All rats exhibited a significantly potentiated startle response in light-noise trials compared with noise-alone trials (*P* < 0.0001), but this was not affected by the treatment (**b**). There was a trend toward TREATMENT effect on the percentage change of cued fear in rats given intra-BNST_dl_ ACSF, OT, or OTA (*P* = 0.0981) (**c**). All rats exhibited a significant potentiation of startle amplitude in noise-alone trials in comparison with pre-test ASR (*P* < 0.0001), but it was not affected by intra-BNST_dl_ injections (**b**). There was no TREATMENT effect on percentage change on non-cued fear (*P* = 0.8993) (**d**), contextual fear (*P* = 0.5384 (**e**), or shock reactivity (*P* *=* 0.8684) (**f**). Comparing discrimination indices from all trials in rats injected with ACSF, OT, and OTA did not show any significant effect of TREATMENT (*P* = 0.1492) (**g**). **h**, **i** The effects of intra-BNST_dl_ administration of ACSF, OT, or OTA on discrimination index (DI) measured in four time blocks during fear memory recall. Each block consists of five noise-alone trials and five light-noise trials, which have been used to calculate DI in each block. There was a significant interaction between TIME and TREATMENT (*P* = 0.0406) and Bonferroni’s post-hoc tests showed a significant difference in the fourth time block between DI of rats injected with ACSF and OT (*P* = 0.0121) as well as rats injected with OT and OTA (*P* = 0.0007, ***P < 0.001, *P < 0.05) (**h**). DI calculated over four time blocks during fear memory recall in negative controls (injections sites outside the BNST_dl_) showed no main effect of TREATMENT (*P* = 0.5828) nor an interaction between TIME and TREATMENT (*P* = 0.9634) (**i**). Cued fear = [(light-noise trials − noise-alone trials)/noise-alone trials] × 100% in context B. Non-cued fear = [(noise-alone trials − pre-test trials)/pre-test trials] × 100% in context B. Contextual fear = [(noise-alone trials − pre-test trials)/pre-test trials] × 100% in context A

All animals exhibited a significantly potentiated startle in light-noise trials compared with noise-alone trials. Two-way RM ANOVA showed a significant main effect of TRIAL TYPE (F(1,50) = 32.01, *P* < 0.0001) but no main effect of TREATMENT (F(2,50) = 0.1656, *P* = 0.8478) and no interaction between TRIAL TYPE and TREATMENT (F(2,50) = 0.7115, *P* = 0.4958; Fig. 5b). Comparison of percentage changes revealed a trend in the TREATMENT effect on cued fear (F(2,50) = 2.433, *P* = 0.0981, ANOVA; Fig. 5c). As high variability was observed in OT-treated rats, we also compared ACSF and OTA-treated groups using unpaired *t*-test, which revealed a trend in the OTA effect on cued fear (*P* = 0.0763).

#### Acquisition of non-cued fear conditioning

Quantitative analysis showed a significant enhancement of ASR in noise-alone trials compared with pre-test trials across all groups. There was a main effect of TRIAL TYPE (pre-test vs. noise-alone, F(1,50) = 22.73, *P* < 0.0001) but no main effect of TREATMENT (F(2,50) = 0.8546, *P* = 0.4316) and no significant interaction (F(2,50) = 0.6216, *P* = 0.5412). When comparing percentage change of non-cued fear with ANOVA, no significant differences were observed between treatment groups (F(2,50) = 0.1063, *P* = 0.8993; Fig. 5d).

#### Acquisition of contextual fear conditioning

Quantitative analysis showed a significant enhancement of ASR in the training context compared with pre-test trials. There was a main effect of TRIAL TYPE (F(1,46) = 12.45, *P* = 0.001) but no main effect of TREATMENT (F(2,6) = 1.35, *P* = 0.2693) and no significant interaction (F(2,46) = 1.096, *P* = 0.3429). Similarly, the mean percent change analysis showed that contextual fear did not differ between treatment groups (F(2,46) = 0.6275, *P* = 0.5384; Fig. 5e).

#### Shock reactivity

The mean shock reactivity during the fear-conditioning session was not different between treatment groups (F(2,50) = 0.1415, *P* = 0.8684; Fig. 5f).

#### Discrimination index

The calculated overall DI was not significantly different between treatment groups (F(2,50) = 1.977, *P* = 0.1492, ANOVA; Fig. 5g). When the DI was calculated over four time blocks, there was no significant main effect of TREATMENT (F(2,50) = 1.92, *P* = 0.1573) or TIME (F(3,150) = 1.47, *P* = 0.2249), but there was a significant interaction between TREATMENT and TIME (F(6,150) = 2.261, *P* = 0.0406, two-way RM ANOVA). Post-hoc comparisons revealed significant differences in the DI in the fourth time block of fear memory recall between ACSF- and OT-treated groups (*t*(200) = 2.91, *P* = 0.0121), as well as between OT and OTA-treated groups (*t*(200) = 3.739, *P* = 0.0007, Bonferroni’s test; Fig. 5h). In the negative controls, the DI over four time blocks revealed no significant main effect of TREATMENT (F(2,42) = 0.5469, *P* = 0.5828) or TIME (F(3,126) = 0.6473, *P* = 0.5861) and there was no significant interaction (F(6,126) = 0.2376, *P* = 0.9634; Fig. 5h**’**).

## Discussion

In the current study, using in vivo microdialysis in freely moving male rats as well as brain-site-specific in vivo pharmacology, we demonstrate that OTR transmission in the BNST_dl_ enables fear learning of cued (signaled, predictable) fear. Using double immunofluorescence labeling of OT and immediate early gene expression cFos, we show that in the hypothalamus, exposure to fear conditioning causes robust activation of OT neurons in the SON and posterior AN.

We previously demonstrated that intra-BNST_dl_ infusion of an OTR antagonist before fear conditioning significantly reduces cued fear recall^12^. In the current study we show that presentation of foot shocks signaled by a discrete cue leads to a significant increase in OT content in BNST_dl_ microdialysates, whereas the presentation of unsignaled foot shocks has no effect. In contrast to previous studies in the PVN^39,40^, central amygdala^41^, and lateral septum^42^, we demonstrate that OT release in the BNST_dl_ is not sensitive to acute stress of FS or foot shocks alone. However, caution needs to be applied when interpreting forced swim results, as high variability of OT content in the BNST_dl_ microdialysates was observed in this experimental group. Previous studies have shown that OT content in the posterior BNST correlate with social discrimination in rats^43^. Although we did not specifically employ a social recognition paradigm, we demonstrate that OT release in the BNST_dl_ is not modulated by SIs with a novel rat. Our results suggest that OT in the BNST_dl_ is released during the acquisition of cued fear, and that OT neurons in the hypothalamus are activated in response to fear conditioning.

Accordingly, we show a significant increase in the percentage of activated OT neurons in the SON and AN in response to signaled and unsignaled foot shocks. Interestingly, the AN has been identified as the main source of OT innervation in the CeA^7^, a critical brain region for acquisition and consolidation of fear memory^44^. We also show activation of OT neurons in the PVN in rats exposed to foot shocks alone, but not cue-signaled foot shocks. Similarly, acute stressors were shown to activate OT neurons in the PVN^45,46^. However, although the PVN sends OT projections to the BNST_dl_^7,30^, we show that OT release in the BNST_dl_ is not evoked by unsignaled foot shocks. Although seemingly contradictory, another population of BNST-projecting hypothalamic OT neurons might modulate acquisition of cued fear, as it has been shown that discrete clusters of OT neurons are highly specialized in their projections and functions^47^. Using retrograde neuronal tracing, we demonstrate that OT neurons from anterior SON and posterior AN also send projections to the BNST_dl_, suggesting they might be specifically involved in facilitating cued fear. Our future studies will identify specific populations of OT neurons in the SON and AN activated by cue-signaled foot shocks. Previously, recall of cued fear conditioning or recall of foot shock alone has been shown to activate OT neurons in the SON and PVN^48^. Notably, in our study, exposure to foot shocks alone generally activated more OT neurons in the hypothalamic nuclei, suggesting that cue presentation may have suppressed the activation of OT neurons. However, our control experiment shows that cue alone does not affect OT neurons’ activation in any of the hypothalamic nuclei. It is therefore possible that OT projection to the BNST_dl_ is selectively involved in strengthening formation of cued fear. Once activated by OT, BNST neurons might have inhibited hypothalamic OT neurons, leading to the observed reduction of their activation. In fact, projection from GABA-ergic neurons in the BNST_dl_ to OT neurons in the PVN has been shown before^49^.

Although we demonstrated that exposure to cue alone does not induce an activation of OT neurons, we could not perform a microdialysis experiment during an exposure to cue alone. Here, microdialysates’ sampling was further complicated by the fact that, once placed in SR-LAB cylindrical enclosures, and not distracted by foot shocks exposure, freely moving rats quickly engage in the consumption and damage of the microdialysis tubing. Nonetheless, as cue alone does not induce any activation of OT neurons, it is unlikely to be that it would induce OT release in the BNST_dl_.

We next investigated the role of OTR transmission in the BNST_dl_ in the acquisition of cued, non-cued, and contextual fear. Structural modification in the OTR antagonist (D-Tyr^2^ instead of Tyr(Me)^2^) renders this compound more selective toward OTR vs. vasopressin receptors in comparison with the antagonist we used before^12^ (also see ref. ^36^). However, in contrast to our previous findings, we show that blocking OTR in the BNST_dl_ before fear conditioning does not significantly reduce cued fear but only induces a trend in cued fear reduction measured in the FPS. However, by calculating DI, we show that infusion of OT potentiates fear discrimination and strengthens fear responses toward cued fear, especially during the later phase of fear recall. Nonetheless, OT-treated group shows high variability of fear responses. Considering that endogenous OT is released during fear acquisition, infusing more exogenous OT might have led to high variability of fear responses. Using selective OTR agonist, (Thr^4^,Gly^7^)-OT, might be a better alternative in the future studies. In previous FPS studies, systemic, but not ICV, administration of OT reduced non-cued fear (background anxiety), without affecting cued or contextual fear^10,11^. Therefore, it is possible that OT dynamically modulates both cued and non-cued fear responses in an opposite manner; it facilitates cued fear in the BNST and reduces non-cued fear responses, overall promoting discrimination between the two. In the BNST_dl_, OT might activate neurons mediating cued fear, which in turn might inhibit neurons responsible for non-cued, sustained fear responses in, or outside the BNST_dl_ (see ref. ^13^). Such dualism was reported in the BNST after auditory fear conditioning, where anterolateral neurons were inhibited, whereas anteromedial BNST neurons were excited in response to CS^+^ presentation^50^. Notably, a similar role of OTR in facilitating fear discrimination has been recently shown in the basolateral amygdala^51^.

Our findings have translational validity for psychiatric disorders in humans. Although healthy controls have greater startle responses to stimuli predicting danger vs. stimuli predicting safety, humans suffering from PTSD demontrate lack of dicrimination between these stimuli^52^. However, startle responses to aversive stimuli adiministered in a predictable manner do not differ between PTSD patients and healthy controls^53^. This highlights the adaptive role of cued/predictable fear, which is necessary to accurately detect and avoid danger. The notion of OT strengthening the adaptive fear and improving the ability to accurately discriminate between danger and safety becomes apparent in animal and human research^13,54^. Notably, a growing number of studies emphasize the role of the BNST in the discrimination between cued (signaled, predictable, phasic) vs. non-cued (unsignaled, unpredictable, sustained) fear^13,25–29,55^. OTR transmission in the BNST_dl_ might play a pivotal role in learning to accurately recognize threats signaled by a dicrete cue.

## Supplementary information

Supplementary Materials

Supplementary Table 1

Supplementary Table 2

## References

[1] Du Vigneaud V (1956). Trail of sulfur research: from insulin to oxytocin. Science (New York, NY).

[2] Jurek B, Neumann ID (2018). The oxytocin receptor: from intracellular signaling to behavior. Physiol. Rev..

[3] Neumann ID, Slattery DA (2016). Oxytocin in general anxiety and social fear: a translational approach. Biol. Psychiatry.

[4] Bale TL, Davis AM, Auger AP, Dorsa DM, McCarthy MM (2001). CNS region-specific oxytocin receptor expression: importance in regulation of anxiety and sex behavior. J. Neurosci..

[5] Ellenbogen MA, Linnen AM, Cardoso C, Joober R (2014). Intranasal oxytocin attenuates the human acoustic startle response independent of emotional modulation. Psychophysiology.

[6] Ring RH (2006). Anxiolytic-like activity of oxytocin in male mice: behavioral and autonomic evidence, therapeutic implications. Psychopharmacology.

[7] Knobloch HS (2012). Evoked axonal oxytocin release in the central amygdala attenuates fear response. Neuron.

[8] Lahoud N, Maroun M (2013). Oxytocinergic manipulations in corticolimbic circuit differentially affect fear acquisition and extinction. Psychoneuroendocrinology.

[9] Guzman YF (2013). Fear-enhancing effects of septal oxytocin receptors. Nat. Neurosci..

[10] Ayers LW, Missig G, Schulkin J, Rosen JB (2011). Oxytocin reduces background anxiety in a fear-potentiated startle paradigm: peripheral vs central administration. Neuropsychopharmacology.

[11] Missig G, Ayers LW, Schulkin J, Rosen JB (2010). Oxytocin reduces background anxiety in a fear-potentiated startle paradigm. Neuropsychopharmacology.

[12] Moaddab M, Dabrowska J (2017). Oxytocin receptor neurotransmission in the dorsolateral bed nucleus of the stria terminalis facilitates the acquisition of cued fear in the fear-potentiated startle paradigm in rats. Neuropharmacology.

[13] Janecek M, Dabrowska J (2018). Oxytocin facilitates adaptive fear and attenuates anxiety responses in animal models and human studies-potential interaction with the corticotropin-releasing factor (CRF) system in the bed nucleus of the stria terminalis (BNST). Cell Tissue Res..

[14] Dabrowska J (2013). Striatal-enriched protein tyrosine phosphatase-STEPs toward understanding chronic stress-induced activation of corticotrophin releasing factor neurons in the rat bed nucleus of the stria terminalis. Biol. psychiatry.

[15] Daniel SE, Rainnie DG (2016). Stress Modulation of Opposing Circuits in the Bed Nucleus of the Stria Terminalis. Neuropsychopharmacology.

[16] Davis M, Walker DL, Miles L, Grillon C (2010). Phasic vs sustained fear in rats and humans: role of the extended amygdala in fear vs anxiety. Neuropsychopharmacology.

[17] Yassa MA, Hazlett RL, Stark CE, Hoehn-Saric R (2012). Functional MRI of the amygdala and bed nucleus of the stria terminalis during conditions of uncertainty in generalized anxiety disorder. J. Psychiatr. Res..

[18] Somerville LH, Whalen PJ, Kelley WM (2010). Human bed nucleus of the stria terminalis indexes hypervigilant threat monitoring. Biol. Psychiatry.

[19] Straube T, Mentzel HJ, Miltner WH (2007). Waiting for spiders: brain activation during anticipatory anxiety in spider phobics. NeuroImage.

[20] Sullivan GM (2004). Lesions in the bed nucleus of the stria terminalis disrupt corticosterone and freezing responses elicited by a contextual but not by a specific cue-conditioned fear stimulus. Neuroscience.

[21] Waddell J, Morris RW, Bouton ME (2006). Effects of bed nucleus of the stria terminalis lesions on conditioned anxiety: aversive conditioning with long-duration conditional stimuli and reinstatement of extinguished fear. Behav. Neurosci..

[22] Gewirtz JC, McNish KA, Davis M (1998). Lesions of the bed nucleus of the stria terminalis block sensitization of the acoustic startle reflex produced by repeated stress, but not fear-potentiated startle. Prog. Neuropsychopharmacol. Biol. Psychiatry.

[23] Hitchcock JM, Davis M (1991). Efferent pathway of the amygdala involved in conditioned fear as measured with the fear-potentiated startle paradigm. Behav. Neurosci..

[24] LeDoux JE, Iwata J, Cicchetti P, Reis DJ (1988). Different projections of the central amygdaloid nucleus mediate autonomic and behavioral correlates of conditioned fear. J. Neurosci..

[25] Goode TD, Maren S (2017). Role of the bed nucleus of the stria terminalis in aversive learning and memory. Learn. Mem..

[26] Gungor NZ, Pare D (2016). Functional heterogeneity in the bed nucleus of the stria terminalis. J. Neurosci..

[27] Duvarci S, Bauer EP, Pare D (2009). The bed nucleus of the stria terminalis mediates inter-individual variations in anxiety and fear. J. Neurosci..

[28] De Bundel D (2016). Dopamine D2 receptors gate generalization of conditioned threat responses through mTORC1 signaling in the extended amygdala. Mol. Psychiatry.

[29] Lange MD (2017). Cannabinoid CB1 receptors in distinct circuits of the extended amygdala determine fear responsiveness to unpredictable threat. Mol. Psychiatry.

[30] Dabrowska J (2011). Neuroanatomical evidence for reciprocal regulation of the corticotrophin-releasing factor and oxytocin systems in the hypothalamus and the bed nucleus of the stria terminalis of the rat: Implications for balancing stress and affect. Psychoneuroendocrinology.

[31] Dumais KM, Bredewold R, Mayer TE, Veenema AH (2013). Sex differences in oxytocin receptor binding in forebrain regions: correlations with social interest in brain region- and sex- specific ways. Horm. Behav..

[32] Tribollet E, Dubois-Dauphin M, Dreifuss JJ, Barberis C, Jard S (1992). Oxytocin receptors in the central nervous system. Distribution, development, and species differences. Ann. N. Y. Acad. Sci..

[33] Veinante P, Freund-Mercier MJ (1997). Distribution of oxytocin- and vasopressin-binding sites in the rat extended amygdala: a histoautoradiographic study. J. Comp. Neurol..

[34] Martinon D, Dabrowska J (2018). Corticotropin-releasing factor receptors modulate oxytocin release in the dorsolateral bed nucleus of the stria terminalis (BNST) in male rats. Front. Neurosci..

[35] Dabrowska J, Nowak P, Brus R (2008). Reactivity of 5-HT1A receptor in adult rats after neonatal noradrenergic neurons’ lesion–implications for antidepressant-like action. Brain Res..

[36] Manning M (2012). Oxytocin and vasopressin agonists and antagonists as research tools and potential therapeutics. J. Neuroendocrinol..

[37] Walker D (2009). Differential effects of the CRF-R1 antagonist GSK876008 on fear-potentiated, light- and CRF-enhanced startle suggest preferential involvement in sustained vs phasic threat responses. Neuropsychopharmacology.

[38] Paxinos, G. & Watson, C. *The Rat Brain in Stereotaxic Coordinates*. 6th ed. (Academic Press, London, 2007).

[39] Bosch OJ, Kromer SA, Brunton PJ, Neumann ID (2004). Release of oxytocin in the hypothalamic paraventricular nucleus, but not central amygdala or lateral septum in lactating residents and virgin intruders during maternal defence. Neuroscience.

[40] Neumann ID (2007). Stimuli and consequences of dendritic release of oxytocin within the brain. Biochem. Soc. Trans..

[41] Ebner K, Bosch OJ, Kromer SA, Singewald N, Neumann ID (2005). Release of oxytocin in the rat central amygdala modulates stress-coping behavior and the release of excitatory amino acids. Neuropsychopharmacology.

[42] Ebner K, Wotjak CT, Landgraf R, Engelmann M (2000). A single social defeat experience selectively stimulates the release of oxytocin, but not vasopressin, within the septal brain area of male rats. Brain Res..

[43] Dumais KM, Alonso AG, Immormino MA, Bredewold R, Veenema AH (2016). Involvement of the oxytocin system in the bed nucleus of the stria terminalis in the sex-specific regulation of social recognition. Psychoneuroendocrinology.

[44] Wilensky AE, Schafe GE, Kristensen MP, LeDoux JE (2006). Rethinking the fear circuit: the central nucleus of the amygdala is required for the acquisition, consolidation, and expression of Pavlovian fear conditioning. J. Neurosci..

[45] Robinson DA (2002). Oxytocin mediates stress-induced analgesia in adult mice. J. Physiol..

[46] Wotjak CT (2001). Forced swimming stimulates the expression of vasopressin and oxytocin in magnocellular neurons of the rat hypothalamic paraventricular nucleus. Eur. J. Neurosci..

[47] Eliava M (2016). A new population of parvocellular oxytocin neurons controlling magnocellular neuron activity and inflammatory pain processing. Neuron.

[48] Zhu L, Onaka T (2002). Involvement of medullary A2 noradrenergic neurons in the activation of oxytocin neurons after conditioned fear stimuli. Eur. J. Neurosci..

[49] Dabrowska, J., Martinon, D., Moaddab, M. & Rainnie D. G. Targeting corticotropin-releasing factor (CRF) projections from the oval nucleus of the BNST using cell-type specific neuronal tracing studies in mouse and rat brain. *J. Neuroendocrinol*. **28** (2016). 10.1111/jne.12442 PMC5362295 27805752

[50] Haufler D, Nagy FZ, Pare D (2013). Neuronal correlates of fear conditioning in the bed nucleus of the stria terminalis. Learn. Mem. (Cold Spring Harb., NY)..

[51] Fam J, Holmes N, Delaney A, Crane J, Westbrook RF (2018). Oxytocin receptor activation in the basolateral complex of the amygdala enhances discrimination between discrete cues and promotes configural processing of cues. Psychoneuroendocrinology.

[52] Jovanovic T (2010). Fear potentiation is associated with hypothalamic-pituitary-adrenal axis function in PTSD. Psychoneuroendocrinology.

[53] Grillon C (2009). Increased anxiety during anticipation of unpredictable aversive stimuli in posttraumatic stress disorder but not in generalized anxiety disorder. Biol. Psychiatry.

[54] Eckstein M (2018). Oxytocin for learning calm and safety. Int. J. Psychophysiol..

[55] Asok A (2018). Optogenetic silencing of a corticotropin-releasing factor pathway from the central amygdala to the bed nucleus of the stria terminalis disrupts sustained fear. Mol. Psychiatry.

