# Supplementary material for: Oxytocin receptors in the dorsolateral bed nucleus of the stria terminalis (BNST) bias fear learning toward temporally predictable cued fear
Source: Transl Psychiatry. 2019 Apr 18;9:140. doi: 10.1038/s41398-019-0474-x (PMC6472379; doi:10.1038/s41398-019-0474-x)
Supplement: Supplementary file 1 — Supplementary Materials [file 41398_2019_474_MOESM1_ESM.docx]

Supplementary materials

**1. Methods and materials**

***1.1 Microdialysis probe implantation***

Microdialysis experiments were performed according to our previously published protocol (1). The two ends of the microdialysis probe were first attached with PE-20 polyethylene tube, followed by flushing and filling of the probe with sterile double-distilled water. Standard stereotaxic procedures were used for unilateral implantation of the microdialysis probe. Rats were implanted with probes containing a U-shaped dialysis membrane (molecular cut-off 18 kDa, Hemophan, Gambro Dialysatoren, Hechingen, Germany; for details, see (2) into left BNST_dl_ (coordinates from Bregma: AP +0.1 mm, ML +3.4 mm, DV -7.25 mm, 15˚ coronal angle). Rats were given an analgesic (5 mg/kg ketoprofen, subcutaneous) prior to the surgery. The surgery was performed with a stereotaxic frame (David Kopf Instruments, USA) using isoflurane anesthesia (E-Z Systems Corporation, Palmer, PA). Small stainless steel screws were inserted into the frontal and parietal bones to secure the probe to the skull using acrylic cement. After the surgery was completed, the outlets of the probe were secured with tape to prevent any damage to the probe until the day of the experiment. Ketoprofen was given again the morning after surgery. Rats were caged individually for 2 days prior to starting the microdialysis experiment. This has been shown to be an optimal timeline as chronic implantation of the microdialysis probe increases the risk of gliosis three days after implantation, which significantly reduces absolute and relative recovery of the microdialysis membrane (3).

***1.2 In vivo microdialysis in freely moving rats***

Rats were placed individually in Plexiglas cages (43 cm x 21 cm x 31 cm) for 30 minutes before connecting the probe to the microinjection pump. Rats were gently restrained and the microdialysis probe was connected to a 3-ml syringe mounted on a microdialysis pump (PHD Ultra Pump, Harvard Apparatus) using a 2-channel spiral tubing (CT-20, Eicom, San Diego, CA, internal volume 4 µl) with connecting Joint Teflon (JT-10, Eicom, San Diego, CA, 4 µl) and a 2-way swivel (Eicom, San Diego, CA). Hence, the total internal volume of inlet and outlet tubing was 16 µl so that any effects of behavioral manipulation could be already observed in the first sample collected post-treatment. This microdialysis study was performed in awake, freely moving rats provided with food and water for the duration of the experiment, except during the behavioral manipulation. The microdialysis probes were perfused with a constant rate of 3.33 µl/min with sterile artificial cerebrospinal fluid (ACSF; composition: 20 mM NaCl, 3.5 mM KCl, 1.1 mM KH_2_PO_4_, 1.3 mM MgCl_2_, 2.5 mM CaCl_2_, 20 mM glucose, 30 mM NaHCO_3_, 0.4 mM ascorbate, 0.8 mM thiourea, 2 mM Na-pyruvate; pH adjusted to 7.4) for 1 h equilibration, during which no samples were collected. After equilibration, three 30-min (100 µl each) baseline samples were collected before any behavioral challenge began. Five more 30-min (100 µl each) microdialysate samples were then collected during and after a behavioral challenge. All the samples were collected in 1.5-ml low-retention Eppendorf tubes placed on ice during collection and immediately frozen after collection on dry ice for storage at -80˚C.

***1.3 The effects of behavioral manipulations on OT content in BNST_dl_ microdialysates***

On any experimental day, treatment groups were counterbalanced such that microdialysis samples from CTRL rats (no behavioral manipulations) were collected in the same room and time as rats exposed to behavioral manipulations. Each rat was individually housed for the duration of the microdialysis experiment.

***1.3.1 Fear-conditioning***

During the fear conditioning, a single LED bulb positioned on the ceiling inside the startle chamber was used as the visual conditioned stimulus (CS). In addition, a grid floor made of stainless steel bars placed inside the enclosures delivered foot shocks as the unconditioned stimulus (US). The presentation and sequence of all stimuli as well as recording of the responses were automatically performed by the SR-LAB software. During habituation (day 1) rats were placed in cylindrical enclosures inside the chambers for 20 min. On the next day (pre-test, day 2), rats were placed in the same enclosures, where after a 5 min acclimation they were presented with 30 startle-eliciting white-noise bursts (WNB, 95 dB, 50 ms, inter-trial-interval 30 s). A high-frequency loudspeaker, mounted 24 cm above the enclosures, provided WNB and background white-noise (70 dB), which continuously played throughout the session. These habituation procedures were designed to reduce novelty-induced stress and to mimic the design used in the behavioral experiments below. Rats were assigned into three treatments groups based on their mean ASR. On day 3, rats underwent stereotaxic surgery for microdialysis probe implantation in the BNST_dl_. They recovered for 2 days prior to fear-conditioning training.

On any given experimental day, three rats were placed individually in microdialysis cages in the behavioral room with the SR-LAB apparatus. Microdialysis tubing was connected as above and three baseline microdialysate samples were collected. After baseline samples collection, two rats were transferred individually to the SR-LAB chambers inside cylindrical enclosures with grid floor conveying foot shocks. A swivel was attached to a small hook in the SR-LAB chamber ceiling to allow free animal movement inside the enclosures.

***1.3.2*** ***Forced swimming (FS)***

Rats were individually placed in Plexiglas microdialysis cages and three baseline samples were collected. Next, rats were placed in Plexiglas tanks filled with water (20°C, up to 40 cm) and forced to swim for 10 min (4). After 10 minutes, rats were removed from the tanks and placed in intermediate cages filled with paper towels for 5 minutes. They were then returned to microdialysis cages and five more microdialysis samples were collected, including the sample collected during FS session.

During forced swim, Plexiglas tanks were placed near the microdialysis cages so that rats could be transferred to the tank filled with water without disconnecting the spiral tubing. The swivel arm with spiral tubing was attached to a trim of Plexiglas tank for the duration of the FS, which allowed for tubing to stay intact and sample collection to remain undisturbed during the event of rats diving inside water tanks. Hence, microdialysis samples were continuously collected during FS session.

***1.3.3 Social interactions (SI)***

Rats interacted without disconnecting the spiral tubing so that microdialysis samples were collected continuously from the experimental rat. After 10 min of social interactions, the novel rat was removed and five more microdialysis samples were collected, including the sample collected during SI.

***1.4. Probe placement***

Following microdialysis and behavioral testing, all rats were euthanized using isoflurane overdose and decapitation. Probes were perfused with Chicago Sky Blue 6B dye (Alfa Aesar, Ward Hill, MA) as a 2% solution in 0.9% saline. The extracted brains were sliced on an SM2000 R sliding microtome (Leica Biosystems, Nussloch, Germany) (50 µm) and photographed to confirm proper placement of the probe. Proper probe or cannula placement met the following criteria: probe tip located in the BNST_dl_ (Bregma + 0.10 mm to - 0.36 mm), above the anterior commissure, below the lateral ventricle and medial to the internal capsule (see examples of proper cannula placements in **Fig. 2A-A’’**, and **Fig. 5A**).

***1.5 Radioimmunoassay for OT***

Frozen dialysates samples were evaporated until dry in a vacuum concentrator (Jouan RC10.10, Thermo Fisher Scientific) with a freeze-dry system (FreeZone 6, LABCONCO). All evaporated microdialysate samples were treated identically. The content of OT in each dialysate was quantified with a highly sensitive (0.1 pg OT/100 µl sample) and selective radioimmunoassay (RIA, minimal affinity for arginine-vasopressin, RIAgnosis, Munich, Germany), as previously described (1, 2, 5, 6). Cross-reactivity of the polyclonal antiserum with [arginine–vasopressin](http://topics.sciencedirect.com/topics/page/Vasopressin) and other related [peptides](http://topics.sciencedirect.com/topics/page/Peptides) was < 0.7%. Intra- and inter-assay coefficients of variation were < 8% and < 11%, respectively.

***2. The effect of fear conditioning on activation of hypothalamic OT neurons***

Here, 15 rats were used to determine whether OT neurons in the hypothalamus are activated in response to fear conditioning. We conducted double immunofluorescence labeling in hypothalamic sections with antibodies against OT and immediate early gene expression, cFos, following fear conditioning.

***2.1 Fear-conditioning and timely perfusions***

Rats were assigned into three treatments groups based on their mean ASR. They were fear-conditioned in SR-LAB chambers. One group of rats (n=5) was exposed to signaled foot-shocks, whereas another group of rats was exposed to un-signaled foot-shocks (n=5). Control rats (n=8) were placed inside the chambers but did not receive cue or shock presentation, another control group (n=3) received cue presentations alone (no foot-shocks).

***2.2 Double immunofluorescence***

Every 3^rd^ brain section from the entire hypothalamus (Bregma -0.60 mm to -2.40 mm) from all 15 rats was processed for double immunofluorescence using anti-OT mouse monoclonal antibody (clone 4G11, 1:5000, MAB5296, Chemicon-Millipore, Billerica, MA), as described before (7), and combined with anti-cFos rabbit polyclonal antibody (sc-52, 1:2000, Santa Cruz Biotechnology, Santa Cruz, CA). Double immunofluorescence protocol was performed as before (7). Briefly, sections were rinsed in phosphate buffer saline (PBS), incubated in 3% normal goat serum (NGS, Thermo Fischer Scientific, Waltham, MA) in 0.5% Triton X-100 (Sigma-Aldrich, St. Louis, MO) in PBS, and incubated for 48 h at 4 °C with the above primary antibodies diluted in 0.5% Triton X-100/PBS solution. Sections were rinsed in PBS and incubated at room temperature for 2 h with specific Alexa Fluor secondary antibodies (1:500, Molecular Probes, Thermo Fischer Scientific, Waltham, MA): Alexa Fluor 488 goat anti-mouse IgG and Alexa Fluor 594 goat anti-rabbit IgG. Following incubation with secondary antibodies, sections were rinsed in PBS and phosphate buffer (PB), mounted on gelatin-coated glass slides and coverslipped using Mowiol-DABCO (Sigma-Aldrich, St. Louis, MO) media, supplementary methods 2.3.2

***2.3 Confocal microscopy and cells counting***

As we observed a considerable variation in OT neurons’ activation in a rostro-caudal manner, in addition to total cell counts, we also analyzed results from anterior to posterior hypothalamic sections, grouping these sections into three AREAS. All hypothalamic sections were first screened for OT neurons’ distribution using an Eclipse Ni-E microscope (Nikon Instruments Inc., Melville, NY) and categorized into three Bregma levels based on anatomical hallmarks (length of the optic track, development of third ventricle, and development of hippocampal formation) and the rat brain atlas (8) into three AREAS (AREA 1, Bregma –0.60 mm to 1.20 mm; AREA 2, -1.32 mm to -1.72 mm; and AREA 3, -1.80 to -2.28 mm). The PVN and AN were divided into AREAS 1-3, whereas SON was divided into AREA 1-2. The PVN and AN were divided into all three AREAS, whereas SON was divided into two AREAS (AREA 1 and 2). Results were additionally analyzed by two-way repeated measures ANOVA with factors TREATMENT and AREA. Where the main or interaction effect was significant, all pairwise post hoc comparisons were made using Bonferroni’s test, ***supplementary Table 2***.

***3. The effects of OT or OTA administration into the BNST_dl_ on FPS***

***3.1 Guide cannula implantation***

Stereotaxic surgery technique was identical to above except that guide cannulas were implanted bilaterally instead of a microdialysis probe (9).

***3.2 Drug administration***

OT (100 ng), OTA (200 ng), or ACSF (all in volume of 0.5 µl per side) was injected bilaterally into the BNST_dl_ through a microinjector (28-gauge, 7 mm length; Plastics One, Roanoke, VA) as described before (9). Doses of OT and OTA were chosen based on previous studies on fear and anxiety in rats (9-13). Rats were assigned based on a second pre-test ASR into three treatment groups. No sample randomization was used. Fear conditioning sessions were performed 10 min after the intra-BNST_dl_ injections.

***3.3 Fear-conditioning and fear recall testing using FPS***

FPS procedures were modified based on previous studies and according to our protocol described in detail before (9, 14-16). After chambers habituation and 2 pre-test sessions (day 1 and 2), on day 3 (fear conditioning), animals were placed in cylindrical enclosures containing a grid floor conveying foot shocks. After 5 min acclimation, animals received 10 presentations of a 3.7 s cue light (CS), each co-terminating with a 0.5 s foot shock (US; 0.5 mA, inter-trial-interval 60–180 s). Background noise was absent during the conditioning session. Enclosures and chambers were cleaned with PREempt RTU disinfectant solution (Virox Technologies, Oakville, Canada) before and after each fear conditioning session (context A). Twenty-four hours later, on day 4, rats were tested for FPS expression (recall test) in context B. After 5 min of acclimation, they were exposed to 50 startle-eliciting WNBs (as above) and levels of cued and non-cued fear were measured. A background white noise of 70 dB continuously played throughout the session. The session consisted of 10 baseline startle trials (excluded from the analysis) followed by an additional 40 trials, with half presented in the presence of the cue light (CS^+^, light-noise) and the other half without the CS (CS^-^, noise-alone) in a pseudorandom order (inter-trial-interval 30 s). To make context A distinct from context B, enclosures and chambers were cleaned with 70% ethanol before and after the fear conditioning sessions (different than in fear conditioning). The grid floor was also removed from the enclosures during cued FPS testing. In addition, a different experimenter from the training session performed the FPS testing session. On day 5, the same rats were tested for contextual fear recall in context A, where after 10 baseline startle trials (excluded from the analysis), startle amplitude was measured during 40 additional CS^-^ trials (noise-alone, inter-trial-interval 30 s).

***3.4 Data analysis***

Startle amplitude was defined as the maximum peak voltage within the first 200 ms after onset of the WNB. Shock reactivity amplitude was recorded during the fear conditioning session and was defined as the maximum peak voltage that occurred during the 500 ms foot shock delivery. Cued, non-cued, and contextual fear, were calculated as percent change scores of startle amplitude based on previous FPS studies (9, 14-16).

***Supplementary Table 1***. Exposure to foot shocks signaled by a cue, but not foot shocks alone, increases OT content in BNST_dl_ microdialysates. In contrast, forced swim stress (FS) or social interactions (SI) do not affect OT release in the BNST_dl_. Data are presented as MEAN ± standard error of mean (SEM) of OT content in BNST_dl_ microdialysates expressed as pg per 100 μl microdialysis sample. There was a significant TREATMENT effect on OT content in BNST_dl_ microdialysates in rats exposed to foot shocks signaled by a cue (*P* = 0.0297, one-way ANOVA).

***Supplementary Table 2***. Fear conditioning activates OT neurons in the hypothalamus. Percentages of OT neurons co-expressing cFos in brain sections from the paraventricular (PVN), supraoptic (SON), as well as accessory nucleus of the hypothalamus (AN) in response to foot shocks signaled by a cue, un-signaled foot shocks, or control conditions are shown as MEAN ± standard error of mean (SEM), * *P*<0.05, ** *P*<0.01, ****P*<0.001, **** *P*<0.0001.

In the PVN, fear conditioning activated OT neurons (F(2,129)=3.142, *P*=0.0465, ANOVA) and post hoc analysis showed a significantly greater percentage of activated OT neurons in rats exposed to foot shocks alone in comparison to CTRL rats (*P*=0.0406). Comparing percentage of activated OT neurons in the PVN across rostro-caudal AREAS for all 3 conditions revealed no significant main effect of TREATMENT (F(2,11)=0.7313, *P*=0.5033), or AREA (F(2,22)=0.1384, *P*=0.8715), and no significant interaction between TREATMENT and AREA (F(4, 22)=1.145, *P*=0.3617, two-way RM ANOVA).

In the SON, fear conditioning activated OT neurons (F(2,82)=31.40, *P*<0.0001, ANOVA). Post hoc analysis revealed a significantly greater percentage of activated OT neurons in rats exposed to foot shocks alone (*P*<0.0001) and foot shocks signaled by a cue (*P*=0.0030) in comparison to CTRL rats. A significant difference was also observed in rats exposed to foot shocks alone vs. foot shocks signaled by a cue (*P*=0.0003).

Comparing percentages of activated OT neurons across AREAS 1-2 of SON for all 3 conditions with a two-way RM ANOVA revealed a significant effect of TREATMENT (F(2,11)=11.21, *P*=0.0022), no significant effect of AREA (F(1,11)=0.05418, *P*=0.8202), and no significant interaction (F(2,11)=1.505, *P*=0.2644). In AREA 1, significantly greater percentage of activated OT neurons was observed in rats exposed to foot shocks alone (*t*(22)=3.526, *P*=0.0057), but not un-signaled foot shocks, compared to CTRL rats (*t*(22)=0.8871, *P*>0.9999). However, there was a significant difference between rats exposed to foot shocks alone vs. foot shocks signaled by a cue within AREA 1 (*t*(22)=2.799, *P*=0.0314). Similarly, within AREA 2, there was a significant effect in rats exposed to foot shocks alone (*t*(22)=4.867, *P*=0.0002), but not in rats exposed to foot shocks signaled by a cue in comparison to CTRL rats (*t*(22)=1.887, *P*=0.2174). There was a significant difference between rats exposed to foot shocks signaled by a cue and un-signaled shocks within AREA 2 (*t*(22)=3.161, *P*=0.0136).

In the AN, a significant TREATMENT effect (F(2,150)=12.62, *P*<0.0001, ANOVA), followed by a post hoc analysis revealed a significantly greater percentage of activated OT neurons in rats exposed to un-signaled (*P*<0.0001) and signaled foot shocks compared to CTRL (*P*=0.0168). Percentages of activated OT neurons did not differ between the two groups (*P*=0.2564).

Comparing percentages of activated OT neurons in the AN across AREAS 1-3 for all three conditions with a two-way RM ANOVA revealed no significant effect of TREATMENT (F(2,11)=2.331, *P*=0.1432), a trend for the AREA (F(2,22)=3.060, *P*=0.0672), and no significant interaction F(4, 22)=1.416, *P*=0.2618).

**5. References**

1. Martinon D, Dabrowska J (2018): Corticotropin-Releasing Factor Receptors Modulate Oxytocin Release in the Dorsolateral Bed Nucleus of the Stria Terminalis (BNST) in Male Rats. *Frontiers in neuroscience*. 12:183.

2. Neumann I, Russell JA, Landgraf R (1993): Oxytocin and vasopressin release within the supraoptic and paraventricular nuclei of pregnant, parturient and lactating rats: a microdialysis study. *Neuroscience*. 53:65-75.

3. Hascup ER, af Bjerken S, Hascup KN, Pomerleau F, Huettl P, Stromberg I, et al. (2009): Histological studies of the effects of chronic implantation of ceramic-based microelectrode arrays and microdialysis probes in rat prefrontal cortex. *Brain research*. 1291:12-20.

4. Dabrowska J, Nowak P, Brus R (2008): Reactivity of 5-HT1A receptor in adult rats after neonatal noradrenergic neurons' lesion--implications for antidepressant-like action. *Brain research*. 1239:66-76.

5. Bosch OJ, Dabrowska J, Modi ME, Johnson ZV, Keebaugh AC, Barrett CE, et al. (2016): Oxytocin in the nucleus accumbens shell reverses CRFR2-evoked passive stress-coping after partner loss in monogamous male prairie voles. *Psychoneuroendocrinology*. 64:66-78.

6. Ross HE, Cole CD, Smith Y, Neumann ID, Landgraf R, Murphy AZ, et al. (2009): Characterization of the oxytocin system regulating affiliative behavior in female prairie voles. *Neuroscience*. 162:892-903.

7. Dabrowska J, Hazra R, Ahern TH, Guo JD, McDonald AJ, Mascagni F, et al. (2011): Neuroanatomical evidence for reciprocal regulation of the corticotrophin-releasing factor and oxytocin systems in the hypothalamus and the bed nucleus of the stria terminalis of the rat: Implications for balancing stress and affect. *Psychoneuroendocrinology*. 36:1312-1326.

8. Paxinos G, Watson C (2009): The rat brain in stereotaxic coordinates. Compact 6th ed. Oxford, UK: Academic Press. Elsevier.

9. Moaddab M, Dabrowska J (2017): Oxytocin receptor neurotransmission in the dorsolateral bed nucleus of the stria terminalis facilitates the acquisition of cued fear in the fear-potentiated startle paradigm in rats. *Neuropharmacology*. 121:130-139.

10. Bale TL, Davis AM, Auger AP, Dorsa DM, McCarthy MM (2001): CNS region-specific oxytocin receptor expression: importance in regulation of anxiety and sex behavior. *The Journal of neuroscience : the official journal of the Society for Neuroscience*. 21:2546-2552.

11. Lahoud N, Maroun M (2013): Oxytocinergic manipulations in corticolimbic circuit differentially affect fear acquisition and extinction. *Psychoneuroendocrinology*. 38:2184-2195.

12. Neumann ID, Slattery DA (2016): Oxytocin in General Anxiety and Social Fear: A Translational Approach. *Biological psychiatry*. 79:213-221.

13. Toth I, Neumann ID, Slattery DA (2012): Central administration of oxytocin receptor ligands affects cued fear extinction in rats and mice in a timepoint-dependent manner. *Psychopharmacology*. 223:149-158.

14. Ayers LW, Missig G, Schulkin J, Rosen JB (2011): Oxytocin reduces background anxiety in a fear-potentiated startle paradigm: peripheral vs central administration. *Neuropsychopharmacology : official publication of the American College of Neuropsychopharmacology*. 36:2488-2497.

15. Missig G, Ayers LW, Schulkin J, Rosen JB (2010): Oxytocin reduces background anxiety in a fear-potentiated startle paradigm. *Neuropsychopharmacology : official publication of the American College of Neuropsychopharmacology*. 35:2607-2616.

16. Walker D, Yang Y, Ratti E, Corsi M, Trist D, Davis M (2009): Differential effects of the CRF-R1 antagonist GSK876008 on fear-potentiated, light- and CRF-enhanced startle suggest preferential involvement in sustained vs phasic threat responses. *Neuropsychopharmacology : official publication of the American College of Neuropsychopharmacology*. 34:1533-1542.
