# Supplementary material for: Oxytocin receptors in the dorsolateral bed nucleus of the stria terminalis (BNST) bias fear learning toward temporally predictable cued fear
Source: Transl Psychiatry. 2019 Apr 18;9:140. doi: 10.1038/s41398-019-0474-x (PMC6472379; doi:10.1038/s41398-019-0474-x)
Supplement: Supplementary file 2 — Supplementary Table 1 [file 41398_2019_474_MOESM2_ESM.docx]

| CONDITION  OT pg/100 μl  MEAN ± SEM | Baseline | 30 min | 60 min | 90 min | 120 min | 150 min |
| --- | --- | --- | --- | --- | --- | --- |
| CTRL FPS (n = 7) | 1.19 ± 0.05 | 1.16 ± 0.08 | 1.18 ± 0.04 | 1.21 ± 0.11 | 1.18 ± 0.04 | 1.15 ± 0.09 |
| SHOCK (n = 6) | 1.20 ± 0.12 | 1.22 ± 0.19 | 1.18 ± 0.18 | 1.22 ± 0.15 | 1.26 ± 0.15 | 1.19 ± 0.16 |
| SHOCK + CUE (n = 8) | 1.06 ± 0.06 | 1.41 ± 0.14 | 1.29 ± 0.11 | 1.12 ± 0.08 | 1.15 ± 0.07 | 1.08 ± 0.09 |
| CTRL FS (n = 10) | 1.06 ± 0.08 | 1.18 ± 0.19 | 0.92 ± 0.04 | 0.96 ± 0.12 | 0.92 ± 0.06 | 0.90 ± 0.13 |
| FS (n = 8) | 1.01 ± 0.10 | 1.11 ± 0.15 | 0.93 ± 0.11 | 0.93 ± 0.10 | 1.07 ± 0.15 | 0.89 ± 0.11 |
| CTRL SI (n = 8) | 1.02 ± 0.04 | 0.90 ± 0.07 | 0.99 ± 0.09 | 0.89 ± 0.08 | 0.95 ± 0.11 | 0.82 ± 0.05 |
| SI (n = 6) | 1.08 ± 0.08 | 0.89 ± 0.10 | 1.11 ± 0.09 | 0.94 ± 0.06 | 1.07 ± 0.11 | 1.09 ± 0.09 |
