# Supplementary material for: Oxytocin receptors in the dorsolateral bed nucleus of the stria terminalis (BNST) bias fear learning toward temporally predictable cued fear
Source: Transl Psychiatry. 2019 Apr 18;9:140. doi: 10.1038/s41398-019-0474-x (PMC6472379; doi:10.1038/s41398-019-0474-x)
Supplement: Supplementary file 3 — Supplementary Table 2 [file 41398_2019_474_MOESM3_ESM.docx]

|  |  | All AREAS | AREA 1 | AREA 2 | AREA 3 |
| --- | --- | --- | --- | --- | --- |
| PVN | Control | 5.43 ± 2.11 | 4.92 ± 3.76 | 4.61 ± 3.11 | 8.90 ± 5.49 |
|  | Shock alone | 13.69 ± 2.73* | 17.21 ± 5.67 | 19.54 ± 10.79 | 7.13 ± 3.73 |
|  | Shock and cue | 9.64 ± 2.33 | 11.46 ± 5.27 | 6.62 ± 2.85 | 11.24 ± 8.73 |
| SON | Control | 3.81 ± 0.79 | 5.60 ± 2.08 | 1.78 ± 0.96 | N/A |
|  | Shock alone | 22.03 ± 2.38**** | 24.69 ± 5.09** | 28.12 ± 5.05*** | N/A |
|  | Shock and cue | 11.91 ± 1.60** | 10.40 ± 3.01 | 11.99 ± 2.84 | N/A |
| AN | Control | 4.69 ± 1.57 | 8.29 ± 7.66 | 2.57 ± 0.83 | 3.58 ± 2.90 |
|  | Shock alone | 18.95 ± 2.14**** | 12.79 ± 4.45 | 15.29 ± 4.60 | 24.69 ± 7.02 |
|  | Shock and cue | 13.58 ± 2.75* | 10.52 ± 6.24 | 12.83 ± 4.32 | 26.24 ± 8.06 |
